# Supplementary material for: Stretchable and Conductive Cellulose/Conductive Polymer Composite Films for On-Skin Strain Sensors
Source: Materials (Basel). 2022 Jul 19;15(14):5009. doi: 10.3390/ma15145009 (PMC9317044; doi:10.3390/ma15145009)
Supplement: Supplementary file 1 [file materials-15-05009-s001.zip › materials-1797317-supplementary.pdf]

Supporting Information

Stretchable and Conductive Cellulose/Conductive Polymer Composite Films for On-Skin Strain Sensors

Joo Won Han [1], Jihyun Park [2], Jung Ha Kim [2], Siti Aisyah Nurmaulia Entifar [2], Ajeng Prameswati [2], Anky Fitrian Wibowo [2], Soyeon Kim [3], Dong Chan Lim [3], Jonghee Lee [4], Myoung-Woon Moon [5], Min-Seok Kim [5] and Yong Hyun Kim [2,6,*]

[1] Industry-University Cooperation Foundation, Pukyong National University, Busan 48513, Korea; hanjoo1020@naver.com
[2] Department of Smart Green Technology Engineering, Pukyong National University, Busan 48513, Korea; ds3dem@naver.com (J.P.); lagamuffin@naver.com (J.H.K.); nurmauliaentifar29@gmail.com (S.A.N.E.); ajengprameswati97@gmail.com (A.P.); ankiz118248@gmail.com (A.F.W.)
[3] Surface Technology Division, Korea Institute of Materials Science (KIMS), Changwon 51508, Korea; kimso1965@kims.re.kr (S.K.); dclim@kims.re.kr (D.C.L.)
[4] Department of Creative Convergence Engineering, Hanbat National University, Daejeon 34158, Korea; jonghee.lee@hanbat.ac.kr
[5] Department of Materials and Life Science Research Division, Korea Institute of Science and Technology, Seoul 02792, Korea; mwmoon@kist.re.kr (M.-W.M.); nanostructures@kist.re.kr (M.-S.K.)
[6] School of Electrical Engineering, Pukyong National University, Busan 48513, Korea
* Correspondence: yhkim113@pknu.ac.kr

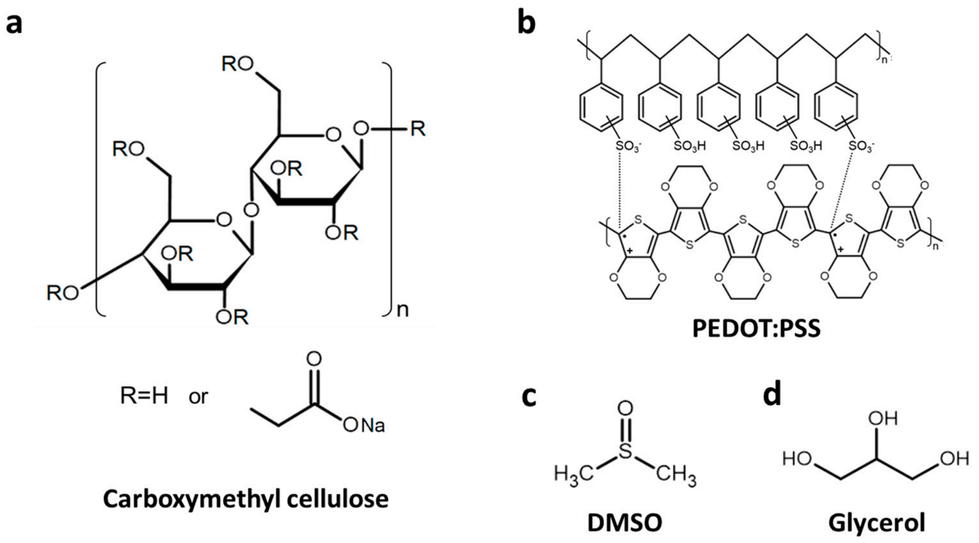

Figure S1. Chemical structures of (**a**) carboxymethyl cellulose (CMC), (**b**) PEDOT:PSS, (**c**) DMSO, and (**d**) glycerol.

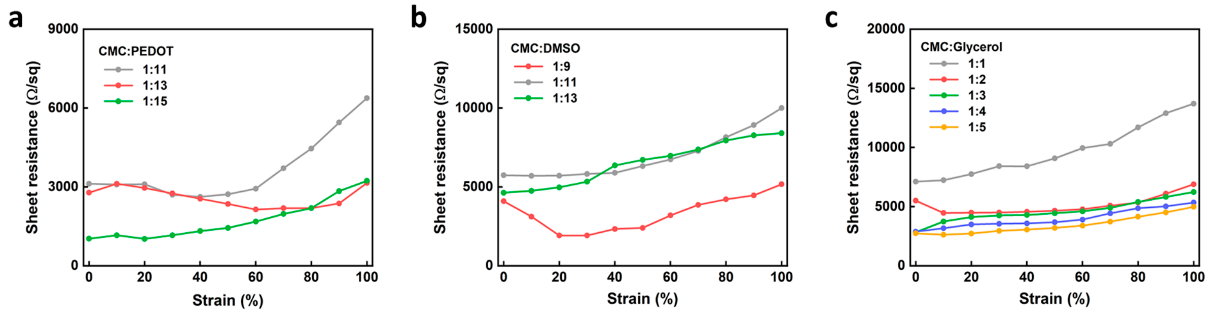

Figure S2. Sheet resistances of CMC-PEDOT:PSS films as a function of ratios of CMC to (**a**) PEDOT:PSS, (**b**) DMSO, and (**c**) glycerol.

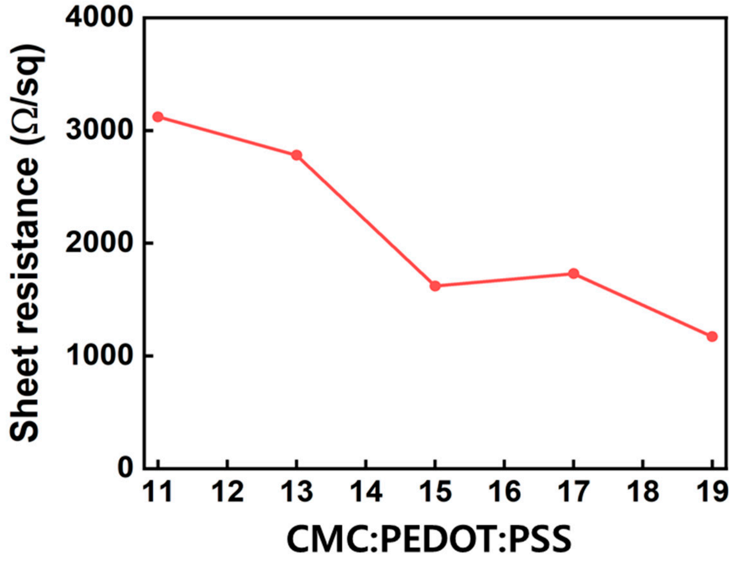

Figure S3. Sheet resistance of the CMC-PEDOT:PSS film (CMC:DMSO=1:11 and CMC:glycerol=1:5) as a function of the ratio of CMC to PEDOT:PSS.

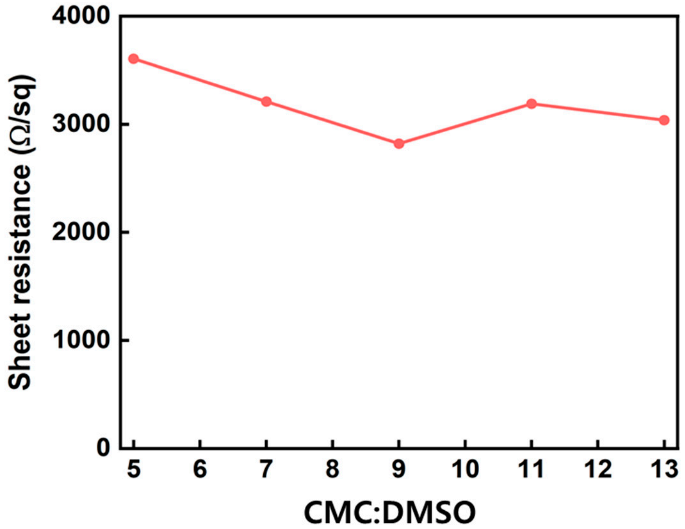

Figure S4. Sheet resistance of CMC-PEDOT:PSS film (CMC:PEDOT:PSS=1:13 and CMC:glycerol=1:2) as a function of the ratio of CMC to DMSO.

Table S1. Cellulose-PEDOT-based composites and their electrical conductivities.

Samples	Flexible or Stretchable	Electrical conductivity (S/cm)	Ref.
Bacterial cellulose (BC) fiber/AuNPs/PEDOT:PSS	Flexible	16.65 ± 1.274	[1]
Cellulose nanofibers(CNFs)/PEDOT:PSS/PPy (CNF/PEDOT:PSS)	Flexible	10.55 (2.58)	[2]
α-Cellulose/PEDOT:PSS/MWCNT (α-Cellulose/PEDOT:PSS)	Flexible	300 (30)	[3]
cellulose nanofiber (CNFs)/PEDOT:PSS	Flexible	22.6	[4]
PEDOT:PSS/CNF nanopaper	Flexible	66.67	[5]
Polythiophene-Derivative Cellulose	Flexible	0.5-0.8	[6]
PEDOT/sulfated cellulose (CS)	Flexible	5.76×10^{-3}	[7]
PVA/Gly-CNC/PVP/PEDOT	Stretchable	$1.73\times10^{-2} \pm 0.099$	[8]
Carboxymethyl cellulose/Lab-synthesized PEDOT:PSS	Stretchable	9.7×10^{-3}	This work

References

1. Khan, S.; Ul-Islam, M.; Ullah, M.W.; Israr, M.; Jang, J.H.; Park, J.K. Nano-Gold Assisted Highly Conducting and Biocompatible Bacterial Cellulose-PEDOT:PSS Films for Biology-Device Interface Applications. *Int. J. Biol. Macromol.* **2018**, *107*, 865–873, doi:10.1016/j.ijbiomac.2017.09.064.

2. Lay, M.; Pèlach, M.À.; Pellicer, N.; Tarrés, J.A.; Bun, K.N.; Vilaseca, F. Smart Nanopaper Based on Cellulose Nanofibers with Hybrid PEDOT:PSS/Polypyrrole for Energy Storage Devices. *Carbohydr. Polym.* **2017**, *165*, 86–95, doi:10.1016/j.carbpol.2017.02.043.

3. Zhao, D.; Zhang, Q.; Chen, W.; Yi, X.; Liu, S.; Wang, Q.; Liu, Y.; Li, J.; Li, X.; Yu, H. Highly Flexible and Conductive Cellulose-Mediated PEDOT:PSS/MWCNT Composite Films for Supercapacitor Electrodes. *ACS Appl. Mater. Interfaces* **2017**, *9*, 13213–13222, doi:10.1021/acsami.7b01852.

4. Ko, Y.; Kim, D.; Kim, U.J.; You, J. Vacuum-Assisted Bilayer PEDOT:PSS/Cellulose Nanofiber Composite Film for Self-Standing, Flexible, Conductive Electrodes. *Carbohydr. Polym.* **2017**, *173*, 383–391, doi:10.1016/j.carbpol.2017.05.096.

5. Du, H.; Zhang, M.; Liu, K.; Parit, M.; Jiang, Z.; Zhang, X.; Li, B.; Si, C. Conductive PEDOT:PSS/Cellulose Nanofibril Paper Electrodes for Flexible Supercapacitors with Superior Areal Capacitance and Cycling Stability. *Chem. Eng. J.* **2022**, *428*, 131994, doi:10.1016/j.cej.2021.131994.

6. González, F.; Tiemblo, P.; Hoyos, M. In-Situ Approaches for the Preparation of Polythiophene-Derivative Cellulose Composites with High Flexibility and Conductivity. *Appl. Sci.* **2019**, *9*, doi:10.3390/app9163371.

7. Horikawa, M.; Fujiki, T.; Shirosaki, T.; Ryu, N.; Sakurai, H.; Nagaoka, S.; Ihara, H. The Development of a Highly Conductive PEDOT System by Doping with Partially Crystalline Sulfated Cellulose and Its Electric Conductivity. *J. Mater. Chem. C* **2015**, *3*, 8881–8887, doi:10.1039/c5tc02074c.

8. Lee, Y.; Choi, H.; Zhang, H.; Wu, Y.; Lee, D.; Wong, W.S.; Tang, X.S.; Park, J.; Yu, H.; Tam, K.C. Sensitive, Stretchable, and Sustainable Conductive Cellulose Nanocrystal Composite for Human Motion Detection. *ACS Sustain. Chem. Eng.* **2021**, *9*, 17351–17361, doi:10.1021/acssuschemeng.1c06741.
